# The C/C Genotype of rs1231760 in *RGS2* Is a Risk Factor for the Progression of *H. pylori*-Positive Atrophic Gastritis by Increasing *RGS2* Expression

**DOI:** 10.3390/diagnostics14222563

**Published:** 2024-11-15

**Authors:** Naoyuki Yamaguchi, Takuki Sakaguchi, Jing-Jing Wei, Yuna Tazoe, Tatsuo Inamine, Daisuke Fukuda, Ken Ohnita, Tatsuro Hirayama, Hajime Isomoto, Kayoko Matsushima, Kazuhiro Tsukamoto

**Affiliations:** 1 Department of Gastroenterology and Hepatology, Graduate School of Biological Sciences, Nagasaki University, 1-7-1 Sakamoto, Nagasaki 852-8501, Japan; 2 Department of Gastroenterology and Nephrology, Faculty of Medicine, Tottori University, 36-1 Nishi-Cho, Yonago 683-8504, Japan; 3 Department of Endoscopy, The First Affiliated Hospital of Fujian Medical University, Fuzhou 350004, China; 4 Department of Pharmacotherapeutics, Graduate School of Biomedical Sciences, Nagasaki University, 1-7-1 Sakamoto, Nagasaki 852-8501, Japan; 5 Department of Surgical Oncology, Graduate School of Biological Sciences, Nagasaki University, 1-7-1 Sakamoto, Nagasaki 852-8501, Japan; 6 Fukuda Yutaka Surgical Clinic, 3-5 Hamaguchi-machi, Nagasaki 852-8107, Japan; 7 Shunkaikai Inoue Hospital, 6-12 Takara-machi, Nagasaki 850-0045, Japan

**Keywords:** *RGS2*, atrophic gastritis, rs1231760

## Abstract

Background: Chronic gastritis caused by *Helicobacter pylori* (*H. pylori*) infection can progress to gastric cancer through atrophic gastritis (AG). The risk of gastric cancer increases with the progression of AG. Therefore, investigating the risk factors for the progression of AG is important. Methods: Using the GTEx and GEO databases, we extracted thirty-four candidate genes involved in the progression of AG. Then, with in silico analysis using HaploReg v4.1 and JASPAR (Matrix ID: MA0113.3), we extracted rs1231760 of *RGS2* as a key single-nucleotide polymorphism (SNP) that could be involved in the functional change in the candidate gene. A correlation analysis between the selected SNP and AG in 200 *H. pylori*-positive and 302 *H. pylori*-negative participants was conducted. For functional analysis of the SNP, a dual-luciferase assay using reporter plasmids with a major or minor allele sequence was carried out. Results: The frequency of the C/C genotype of rs1231760 was higher in the AG group than in the non-AG group (*p* = 0.0471). Functional analysis showed that the transcriptional activities were higher at the dexamethasone-stimulating C allele than at the others (*p* < 0.05). Conclusions: The C/C genotype of rs1231760 in *RGS2* could be a biomarker of high-risk *H. pylori*-positive AG because of an increase in RGS2 expression.

## 1. Introduction

*Helicobacter pylori* (*H. pylori*), which is defined as a “definite carcinogen” for gastric cancer by the International Agency for Research on Cancer in 1994 [1], is a Gram-negative bacillus that commonly colonizes the human stomach. It is transmitted orally and survives by neutralizing gastric acid with self-produced urease [2,3]. One of the most well-known virulence of *H. pylori* is cytotoxin-associated gene A (CagA). CagA is a 120–140 kDa protein that affects the expression of cellular signaling proteins. CagA is degraded by the host’s autophagy system, which degrades the unnecessary cell components to maintain homeostasis.

According to the Correa cascade [4], chronic infection with *H. pylori* leads to atrophic gastritis (AG), intestinal metaplasia, and, finally, gastric cancer, which is the fifth most common cancer and the third most common cause of cancer mortality [5]. However, not all *H. pylori*-positive patients progress to AG, intestinal metaplasia, or gastric cancer. It is estimated that the progression risk of AG to gastric cancer ranges from 0.1% to 0.3% per year or 1–3% of infected individuals [6,7]. This risk is known to increase with the severity of AG [8]. The risk of gastric cancer in patients with *H. pylori* infection with severe AG is 4.9 times higher than that in *H. pylori*-negative patients without AG [9]. Even after eradication therapy, the risks of gastric cancer increase with the severity of AG before the eradication therapy [10]. Generally, the longer the duration of *H. pylori* infection, the more severe the AG [11]. Therefore, elderly patients often experience severe AG. However, in clinical practice, there are some older patients who are *H. pylori*-positive who do not have severe AG, whereas some children or infants have severe AG [12,13]. These clinical cases indicate that host factors affect the development of AG and the progression to gastric cancer.

We have previously reported that there are correlations between single-nucleotide polymorphisms (SNPs) in autophagy-related genes (*ATGs*) and the severity of AG [14,15,16]. These studies were based on the signaling pathways through which the host gastric mucosal cell degrades CagA. However, the relationship between the SNPs in other genes and AG has not been fully elucidated. In this study, we aimed to investigate the other unpredictable genes that are associated with the progression of AG using the gene expression data from the GTEx portal [17] and GEO [18].

## 2. Materials and Methods

### 2.1. Selection of Candidate Genes and Their SNPs

Using the GTEx Portal, we made a list of 2000 candidate genes expressed in the stomach for which expression is strongly correlated with SNP genotypes (List A). Furthermore, we made List B, which was a list of genes that show significantly different levels of expressions (*p* < 0.05) in patients who are *H. pylori*-positive with and without AG [19] (GEO accession: GSE27411), and List C was a list of genes of patients with and without severe gastritis [20] (GEO accession: GSE60662) using GEO. These SNPs in genes selected from both the GTEx Portal and GEO were expected to alter the gene expressions in the stomach and promote *H. pylori*-induced gastritis progression. Therefore, we extracted the genes that matched these three lists. From these genes, we selected genes with either consistently increasing or decreasing expression in both atrophic and severe gastritis.

Next, using HaploReg v4.1 [21] and JASPAR (Matrix ID: MA0113.3) [22], an SNP for each candidate gene that met all of the following three conditions was selected for further analysis: (1) transcription-activated histone modification of the flanking sequence including the SNP was apparent in the gastric mucosa; (2) transcription factor binding to the flanking sequence including the SNP was reported; and (3) the target transcription factor sequence in the flanking sequence including the SNP was modified by the SNP.

### 2.2. Study Participants

This study included 502 participants who underwent esophagogastroduodenalendoscopy at the Fukuda Surgery Clinic for their health check-ups. We divided the participants into two groups, 200 participants who were *H. pylori*-positive and 302 who were *H. pylori*-negative, using the *H. pylori* antibody (E-plate Eiken *H. pylori* antibody II; Eiken Chemical, Tokyo, Japan). After a thorough explanation, all the participants signed the informed consent, and blood was collected for genetic analysis. This study was approved by the Human Genetic Analysis Research Institutional Review Board of Nagasaki University (No. 120111).

### 2.3. Classification of Gastric Mucosal Atrophy

AG was evaluated using the pepsinogen (PG) method [23], in which the levels of two types of serum pepsinogen are measured. Cases with a PG I ≤ 70 μg/L and a PG I/II ratio ≤3.0 were considered as AG. On the other hand, cases were considered non-AG when the above conditions were not met.

### 2.4. Genotype Determination

Following the manufacturer’s protocol, we extracted DNA from participants’ peripheral blood using a NucleoSpin Blood kit (Takara, Shiga, Japan). We determined the genotype of the target SNP using the polymerase chain reaction (PCR)–high-resolution melting (HRM) method with unlabeled probes. The method was as follows: First, the peripheral region, including the SNP, was amplified by PCR using a T100 Thermal Cycler (Bio-Rad, Hercules, CA, USA). The total PCR reaction volume (15 μL) comprised 10 ng of genome DNA, 1× Go Taq Colorless Master Mix (Promega, Madison, WI, USA), 0.06 μM forward primer, and 0.3 μM reverse primer (Table S1). PCR was conducted by denaturing at 95 °C for 2 min, followed by 50 reaction cycles (30 s at 95 °C, 30 s of annealing at 60 °C, and 30 s extension at 72 °C), and a final 5 min extension at 72 °C. The PCR products were analyzed by HRM using a LightCycler 480 II (Roche Diagnostics, Basel, Switzerland). The total HRM reaction volume (15 μL) comprised 10 μL of PCR products, 0.3 μM probe, and 2 μM SYTO9 (Invitrogen Life Technologies, Carlsbad, CA, USA). The probe was a 34-base oligonucleotide that formed a complementary sequence to the major allele of the SNP. Three mismatched bases were added to the 3′ terminal to prevent the extension of the probe itself. For the HRM analysis, the reaction solution was heated at 95 °C for 1 min and 40 °C for 1 min. After that, the amount of luminescence change was measured from 55 °C to 85 °C. The melting curves of the PCR products were analyzed using LightCycler 480 Gene-Scanning software version 1.5 (Roche Diagnostics). The substances for which a melting curve was obtained at high temperatures were considered homozygous for the major allele, those for which melting curves were obtained at low temperatures were considered homozygous for the minor allele, and those for which melting curves were obtained on both sides were considered heterozygous.

### 2.5. Preparation of Reporter Plasmid DNA

To measure transcriptional activity, 50 bp insert sequences, including the recognition sequences for the target rs1231760 SNP (T > C) and recognition sequences for the glucocorticoid receptor (GR), were inserted upstream from the promotor using a pGL3-Promoter Vector (5010 bp; Promega) (Table S2). Cloning at the *Sac* I-*Nhe* I restriction enzyme site was conducted using a Rapid DNA Ligation Kit (Roche Diagnostics) to produce the reporter plasmid. The sequences of the resulting plasmid DNA were confirmed by DNA direct sequencing.

### 2.6. Transfection and Dual-Luciferase Assay

The A549 human alveolar basal epithelial adenocarcinoma cell line (DS Pharma Biomedical, Osaka, Japan) was cultured in high-glucose D-MEM (FUJIFILM Wako Pure Chemical Industries, Osaka, Japan) containing 10% fetal bovine serum (Thermo Fisher Scientific, Waltham, MA, USA) and 1% Antibiotic Antimycotic Solution (GE Healthcare Japan, Tokyo, Japan) at 37 °C under 5% CO_2_. A549 cells were seeded (5.0 × 10^4^ cells/well) onto 24-well plates in 500 μL of D-MEM.

To investigate gene expression in the A549 cell line, mRNA was extracted using RNAzol RT (Molecular Research Center, Cincinnati, OH, USA), and cDNA was produced by reverse transcription. The total reverse-transcription reaction volume (10 μL) comprised 350 ng of mRNA and 2 μL of TAKARA 5× Prime Script RT master mix. The mixture was reacted in a T100 Thermal Cycler at 37 °C for 15 min and 85 °C for 5 s, and the cDNA was analyzed by quantitative PCR using a LightCycler 480 II. The total PCR reaction volume (15 μL) comprised 15 ng of RNA, 7.5 μL of 1X KAPA SYBR FAST qPCR Master Mix (Kapa Biosystems, Cape Town, South Africa), 200 nM forward primer, and 200 nM reverse primer (Table S3). The quantitative PCR reaction was conducted by thermal denaturing at 95 °C for 3 min, followed by 40 reaction cycles (10 s at 95 °C, 20 s of annealing at 60 °C, and 1 s of extension at 72 °C) to amplify the cDNA, and reacting at 95 °C for 5 s and 65 °C for 1 min to produce melting curves.

During transfection using Lipofectamine 3000 Transfection Reagent (Thermo Fisher Scientific), the plasmid DNA was added to the wells at a concentration of 500 ng/well, and, for the purpose of standardization, reporter plasmids containing Nanoluc were also added at 12 ng/well of Opti-MEM (Invitrogen Life Technologies). For transfection, 3 μL of Lipofectamine 3000 reagent and 2 μL of P3000 reagent per 1 μg DNA were mixed and left at 25 °C for 10 min. Then, 50 μL of the mixture was instilled per well. Six hours after transfection, cells were stimulated with or without 50 nM dexamethasone (Dex) and incubated for an additional 42 h.

The transfected cells were harvested and treated with 100 μL of Passive Lysis Buffer (PLB, Promega) to extract proteins. A Nano-Glo Dual-Luciferase Reporter Assay (Promega) was used as the test reagent, and FLUOstar OPTIMA (BMG LABTECH, Offenburg, Germany) was used for measurement. We added 30 µL of ONE-Glo EX Luciferase Assay Reagent to 30 μL of PLB supernatant and measured Firefly luminescence. Then, we added 30 µL of NanoDLR Stop&Glo Reagent (Promega) and measured the Nanoluc luminescence. The F/N ratio was calculated as the ratio of Firefly to Nanoluc luminescence. A pGL3-promoter that did not include the insert sequence was used as an experimental control.

### 2.7. Statistical Analyses

The correlation of the SNP with disease progression was determined by univariate logistic regression analysis using JMP Pro 13 (SAS Institute Japan, Tokyo, Japan). Statistical analyses of sex and genotypes between the AG and non-AG groups were conducted using a chi-square test in Prism 5 (GraphPad Software, La Jolla, CA, USA). A comparison of age between the AG and non-AG groups was carried out using a Mann–Whitney U test in Prism 5. Levels of serum PG were compared among genotypes by a Mann–Whitney U test. The dual-luciferase assay data were analyzed by one-way analysis of variance (ANOVA), followed by a Tukey test in Prism 5, and the results are expressed as the mean ± standard deviation (SD). Statistical significance was set at *p* < 0.05.

## 3. Results

### 3.1. The Clinical Characteristics of the Participants

The mean age of the participants was 53.0 ± 10.9 years, with a sex distribution of 217 men (43.2%) and 285 women (56.8%). Participants in the AG group were significantly older than those in the non-AG group (58.7 ± 10.05 years vs. 51.5 ± 10.61 years, *p* = 2.01 × 10^−10^). However, no significant difference was observed in the sex distribution between the two groups (male/female: 41/64 vs. 176/221, *p* = 0.033).

In the participants who were *H. pylori*-positive, those with AG were older than those who were non-AG (59.2 ± 9.52 vs. 54.9 ± 10.93, *p* = 0.002), and there were no sex differences between these two groups (Table 1). On the other hand, in the *H. pylori*-negative group, there were no differences in age or sex between the AG and non-AG groups (Table 2).

### 3.2. Candidate Gene Extraction by Transcriptome Data Matching

Gene List A had 2000 genes, and List B and List C contained 2644 and 5285 genes, respectively. Thirty-four genes were contained in these three lists. Among the 34 genes, 15 genes had differential expression in the same directions between gene Lists B and C, as shown in Table 3. Among the genes having some SNPs that were highly linked to gene expressions, the regulator of G-protein signaling 2 (RGS2; OMIM ID: 600861) was identified with HaploReg v4.1. GEO showed that the mRNA expression of this gene in the stomach is increased in both patients with severe gastritis [20] or AG [19] as compared to healthy controls infected with *H. pylori*. The C/C genotype of rs1231760 shows the highest expression of RGS2 in the gastric mucosal cells in the GTEx Portal data. Furthermore, according to JASPAR, the C allele of rs1231760 SNP is necessary for GR binding. Therefore, the rs1231760 SNP of RGS2 was selected for further analysis in this study.

### 3.3. RGS2 rs1231760 SNP Is Associated with Atrophic Gastritis in Patients Who Are H. pylori-Positive 

The correlation analysis of rs1231760 SNP with AG in participants who were *H. pylori*-positive showed that the frequency of the C/C genotype in rs1231760 was higher in those who were *H. pylori*-positive with AG than that in those who were non-AG (*p* = 0.0471) in the minor allele recessive model, as shown in Table 4. On the other hand, this association between rs1231760 and AG was not apparent in patients who were *H. pylori*-negative (Table 5).

### 3.4. PG I Is Significantly Lower in Patients with C/C-Genotype rs1231760 SNP

**Figure 1:**
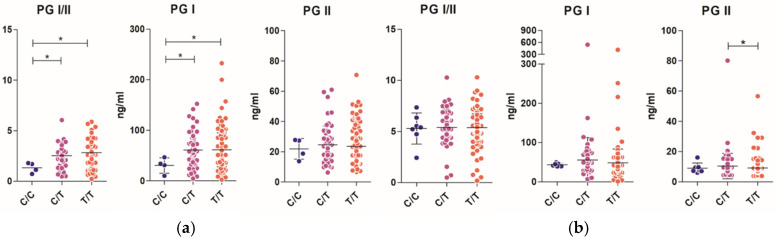
The serum PG I and PG II levels and PG I/II ratio were plotted in participants who were *H. pylori*-positive (**a**) and in *H. pylori*-negative (**b**) with each genotype of rs1231760. * *p* < 0.05, per Mann–Whitney U test.

The PG I level and PG I/II ratio are known to decrease as gastric mucosa atrophy progresses. The participants who were *H. pylori*-positive with the C/C genotype had significantly lower PG I levels than those with other genotypes (C/T or T/T) (*p* < 0.05), as shown in Figure 1a. There was no significant difference in the PG II levels among the three genotypes. Thus, the PG I/II ratio was also lower in participants with the C/C genotype than in those with other genotypes (C/C vs. C/T or C/C vs. T/T, *p* < 0.05) (Figure 1a). There were no differences in participants who were *H. pylori*-negative (Figure 1b).

### 3.5. Transcriptional Activity Is Higher for the C Allele of rs1231760

**Figure 2:**
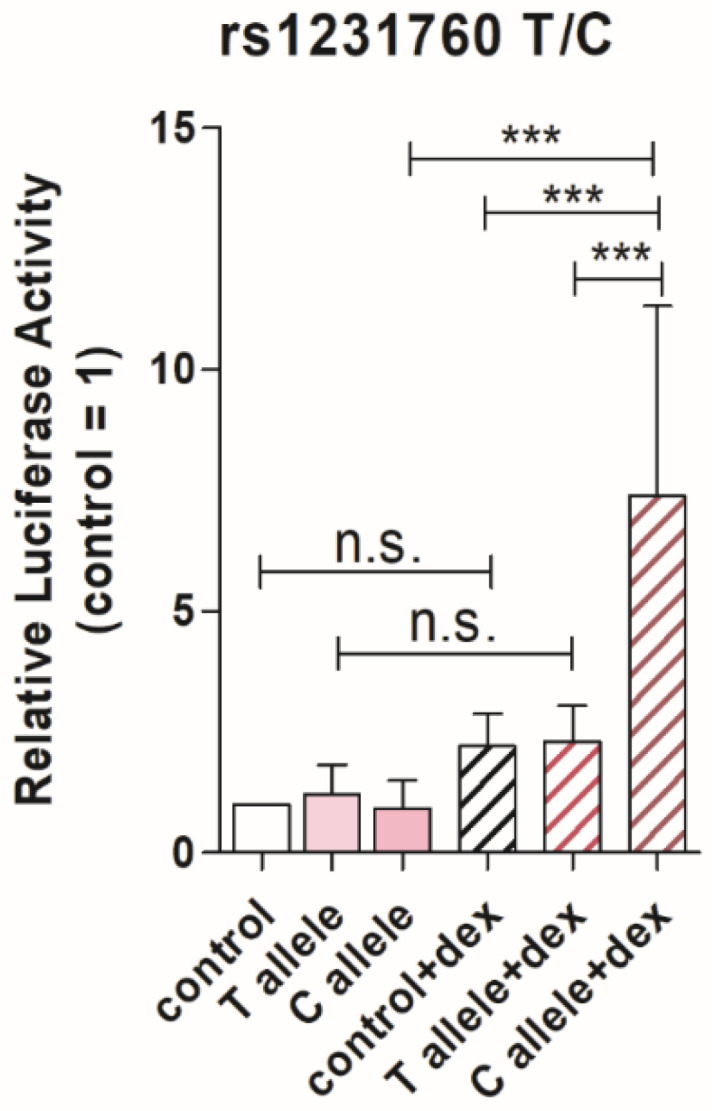
Luciferase activities of T or C allele of rs1231760 in *RGS2* in A549 cells with or without dexamethasone stimulation. To compare the transcriptional activities between the T and C alleles of rs1231760 in RGS2, a Firefly reporter plasmid containing the T or C allele of rs1231760 and GR binding sequence and Nanoluc luciferase plasmid were cotransfected into A549 cells. The Firefly luciferase activity was normalized by that of Nanoluc. Data are presented as the mean ± SD from five independent experiments. *** *p* < 0.001, one-way ANOVA with Tukey’s test.

We confirmed the effect of each allele of rs1231760 in RGS2 on the reporter gene expression in the A549 cells. Transcriptional activities were higher for the dexamethasone-stimulated C allele than those of the control, unstimulating T allele, unstimulating C allele, stimulating control, and stimulating T allele (*p* < 0.001, Figure 2). Without dexamethasone stimulation, there was no difference between the T and C alleles. Dexamethasone stimulation was required to significantly increase transcriptional activity at the only C allele.

## 4. Discussion

In this study, we confirmed the candidate gene, *RGS2*, extracted using the gene expression database from the GTEx Portal and GEO, to be a novel genetic biomarker associated with the progression of AG. *RGS2* has been reported to be related to several cancers, such as breast cancer [24], prostate cancer [25], ovarian cancer [26], and acute myeloid leukemia [27]. Furthermore, Yang et al. reported that those with a high expression of *RGS2* show poor overall survival compared with those with a low expression of *RGS2* in gastric cancer [28]. They also showed that the expression of RGS increased with the increase in clinical stage using immunohistochemical staining [28].

RGS2 is a member of the G protein signal transduction regulator family and regulates G protein activation by activating the Gα subunit GTPase. In gastric cancer, the expression of RGS2 is correlated with the infiltration of 10 types of immune cells [28]. It is suspected that RGS2 contributes to the initiation and progression of gastric cancer by influencing the migration of immune cells [28]. Therefore, in AG, i the high expression of RGS2 could reduce host immunity and cause persistent infection with *H. pylori*. However, there is no report about the function of RGS2 in gastric mucosa. Further functional analysis of RGS2 using gastric mucosa with *H. pylori* infection is required.

Among the SNPs in *RGS2*, the GEO and GTEx data show that the C/C genotype of rs1231760 shows the highest expression of *RGS2* in the gastric mucosal cells. According to JASPAR, which is a database of transcription factor binding profiles, the C allele sequence of rs1231760 is required for the binding activity of the transcription factor GR. Therefore, we examined whether this SNP could be a risk factor for the progression of AG using data from 200 participants who were *H. pylori*-positive and 302 who were *H. pylori*-negative. We found that the C/C genotype of rs1231760 in *RGS2* is a novel genetic factor that is involved in the progression of AG in participants with *H. pylori*. Furthermore, we also found the C/C genotype of rs1231760 is related to lower serum PG I levels and PG I/II ratios than the other genotypes in participants who were *H. pylori*-positive but not in those who were *H. pylori*-negative. PG I is secreted mainly from the chief cells in the fundic mucosa, whereas PG II is secreted from the stomach as a whole [29]. As pointed out in the ABC method [30], which is used as an indicator of AG in Japan, low PG I levels and PG I/II ratio mean the destruction of chief cells in the fundic mucosa. That is, the C/C genotype of rs1231760 in participants who were *H. pylori*-positive increases the risk of AG progression under *H. pylori* infection.

The functional analysis of the rs1231760 SNP was conducted using the A549 cell line, in which GR has been reported to bind to this SNP flanking region under dexamethasone stimulation. The expression of the reporter gene was increased only under the condition of the C allele and dexamethasone. It was reported that cortisol concentrations are increased in those with *H. pylori* [31]. With *H. pylori* infections, the C/C genotype of rs1231760 would increase the expression of *RGS2* due to an increase in cortisol concentrations. As mentioned above, RGS2 expression was reported to be increased in the stomach of patients with atrophy or severe gastritis.

**Figure 3:**
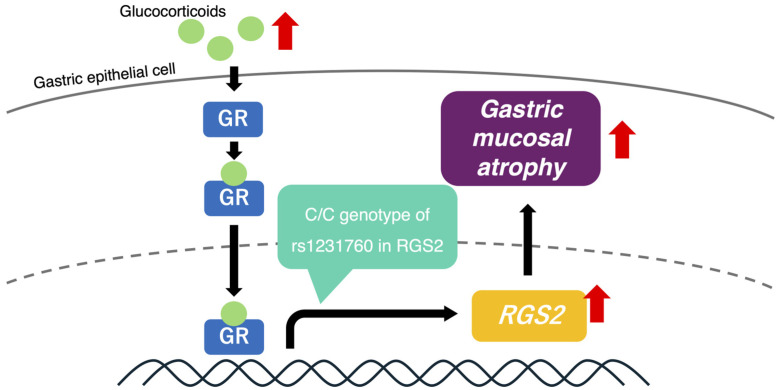
A putative mechanism of gastric mucosal atrophy progression under conditions of increased glucocorticoid concentration. In the stomach of individuals *with H. pylori*, increased glucocorticoid activates GR. In the presence of the C/C-genotype of rs1231760 in *RGS2*, GR could bind to the GR element, including this SNP, thereby activating the transcription of *RGS2* and finally resulting in an increase in the RGS2 levels and progression of gastric mucosal atrophy. Data from GEO showed that the mRNA expression of *RGS2* was increased in both severe and atrophic gastritis as compared to that in controls in the right upper region. Data from GTEx showed that the mRNA expression of *RGS2* was higher in the C/C genotype of rs1231760 in *RGS2* than in other genotypes.

Figure 3 shows a putative model of the progression of AG in individuals who are *H. pylori*-positive and have the C/C genotype of rs1231760 in *RGS2*. The glucocorticoid concentration is increased because of *H. pylori* infection, and glucocorticoid binding to GR increases the expression of *RGS2* in those with the C/C genotype of rs1231760. The mechanism of how *RGS2* causes AG needs to be further explored.

There are several limitations in this study. First, there are several risk factors, for example, tobacco use, high-salt diet, and chronic bile acid reflux, other than age and sex in developing AG [32]. And, there is the hypothesis that moderate alcohol consumption reduces the progression of AG through facilitating the elimination of *H. pylori* [33]. Second, we did not examine the strain of *H. pylori* or the type of CagA or vacuolating cytotoxin A. However, almost all Japanese patients are infected with the East-Asian-type *H. pylori* strain. Third, biopsies for the diagnosis of AG were not conducted. More extensive cohorts evaluated using the OLGA/OLGIM systems are required in the future. However, in this study, we identified the C/C genotype of rs1231760 in *RGS2* as a risk factor for the progression of AG with *H. pylori* infection.

## 5. Conclusions

We used transcriptome databases to identify *RGS2* as a novel candidate gene. The C/C genotype of rs1231760 in *RGS2* is associated with the progression of AG. Furthermore, not only the presence of the C/C genotype of rs1231760 but also an increase in glucocorticoid concentration accelerated the mRNA expression of *RGS2* under *H. pylori* infection. These findings could be helpful when the patients with *H. pylori* are children or when they fail the second eradication therapy because Japanese health insurance does not cover children or third eradication therapy. They could also be helpful when patients risk experiencing side effects, such as antibiotic allergies.

In the future, the mechanisms of how the C/C genotype of rs1231760 increases RGS2 expression and how RGS2 promotes the progression of AG need to be investigated. Those future studies may help prevent the development of gastritis and, ultimately, stomach cancer.

## Figures and Tables

**Table 1:** Clinical characteristics of participants who were *H. pylori*-positive.

Characteristic	Pepsinogen Classification		*p* Value *
	Atrophy (+) (*n* = 94)	Atrophy (−) (*n* = 106)	
Age (year)	59.2 ± 9.52	54.9 ± 10.93	0.002
Sex (male/female)	37/57	50/56	0.266

Age is expressed as mean ± SD. * Mann–Whitney U or chi-square test.

**Table 2:** Clinical characteristics of participants who were *H. pylori*-negative.

Characteristic	Pepsinogen Classification		*p* Value *
	Atrophy (+) (*n* = 11)	Atrophy (−) (*n* = 291)	
Age (year)	55.0 ± 13.83	50.28 ± 10.24	0.174
Sex (male/female)	4/7	126/165	0.763

Age is expressed as mean ± SD. * Mann–Whitney U or chi-square test.

**Table 3:** Altered expression of candidate genes in gastric atrophy and severe gastritis.

Gene	Expression	Function
*UGGT2*	down	Quality control for protein transport out of the endoplasmic reticulum
*ECHDC3*	down	Catalytic enzymes
*SYTL4*	down	Proteins that participate in intracellular membrane trafficking
*HYAL3*	down	Endoglycosidases that degrade hyaluronan
*ZNF85*	down	Transcriptional repressor
*ZNF160*	down	Transcriptional repressor
*PTPN14*	down	Signaling molecules that regulate a variety of cellular processes
*PPP2R3A*	down	Negative control of cell growth and division
*LPIN1*	up	Nuclear transcriptional coactivator
*GNLY*	up	Antimicrobial activity against *Mycobacterium tuberculosis* and other organisms
*RGS2*	up	GTPase-activating proteins
*PEPD*	up	Proline recycling enzyme
*SLC35F2*	up	Transmembrane transporter
*ARSE*	up	Essential enzymes in bone and cartilage matrix composition
*BAK1*	up	Apoptosis-inducing proteins

**Table 4:** Genotype comparison between participants who were *H. pylori*-positive with and without atrophy.

SNP	Genotype	Number of Genotypes		Genetic Model	OR (95% CI)	*p* Value *
		AG *n* = 94 (%)	Non-AG *n* = 106 (%)			
rs1231760	MAF	0.202	0.132	Allele model	0.6007 (0.3523–1.0243)	0.0596
	T/T	60 (63.8)	78 (73.6)			
	C/T	30 (31.9)	28 (26.4)	Dominant model	1.5786 (0.8638–2.8846)	0.1365
	C/C	4 (4.3)	0 (0)	Recessive model	-	0.0471

* Alleles and genotypes were compared with logistic regression analysis.

**Table 5:** Genotype comparison between participants who were *H. pylori*-negative with and without atrophy.

SNP	Genotype	Number of Genotypes		Genetic Model	OR (95% CI)	*p* Value *
		AG *n* = 94 (%)	Non-AG *n* = 106 (%)			
rs1231760	MAF	0.227	0.167	Allele model	1.471 (0.5298–4.082)	0.3947
	T/T	7 (63.6)	200 (68.7)			
	C/T	3 (27.3)	85 (29.2)	Dominant model	1.2559 (0.3587–4.3977)	0.7459
	C/C	1 (9.1)	6 (2.1)	Recessive model	4.750 (0.5216–43.2547)	0.2308

* Alleles and genotypes were compared by logistic regression analysis.

## Data Availability

The datasets generated during the current study are not publicly available due to data sharing not being written in the informed consent.

## Supplementary Materials

The following supporting information can be downloaded at: https://www.mdpi.com/article/10.3390/diagnostics14222563/s1, Table S1: Information for SNP, PCR, and probe-based HRM; Table S2: Information on inserted DNA sequences for target SNP in *RGS2*; Table S3: Information on qPCR of *RGS2* and *NR3C1.*
